# What we need to know about the germ-free animal models

**DOI:** 10.3934/microbiol.2024007

**Published:** 2024-02-06

**Authors:** Fatemeh Aghighi, Mahmoud Salami

**Affiliations:** Physiology Research Center, Institute for Basic Sciences, Kashan University of Medical Sciences, Kashan, I. R. Iran

**Keywords:** Antibiotic–treated animal model, isolated germ-free animal model, gut microbiota, eubiosis, dysbiosis

## Abstract

The gut microbiota (GM), as a forgotten organ, refers to the microbial community that resides in the gastrointestinal tract and plays a critical role in a variety of physiological activities in different body organs. The GM affects its targets through neurological, metabolic, immune, and endocrine pathways. The GM is a dynamic system for which exogenous and endogenous factors have negative or positive effects on its density and composition. Since the mid-twentieth century, laboratory animals are known as the major tools for preclinical research; however, each model has its own limitations. So far, two main models have been used to explore the effects of the GM under normal and abnormal conditions: the isolated germ-free and antibiotic-treated models. Both methods have strengths and weaknesses. In many fields of host-microbe interactions, research on these animal models are known as appropriate experimental subjects that enable investigators to directly assess the role of the microbiota on all features of physiology. These animal models present biological model systems to either study outcomes of the absence of microbes, or to verify the effects of colonization with specific and known microbial species. This paper reviews these current approaches and gives advantages and disadvantages of both models.

## 1. Introduction

For a long time it was thought that the contents of the bowel are simply waste products, disregarding a huge and vital community inhabiting in different organs of the body [1]. The role of gut microbes to human health was first appreciated by Elie Metchnikov in the early 20^th^ century [2]. Now, after several decades, it seems that not only, can we not ignore our intestinal guests, but we also intensively need these microorganisms for a healthy life [3],[4]. This population of microorganisms is named the “gut microbiota” (GM). For a long time, it was assumed that the microorganisms could affect only the gastrointestinal tract. However, it is now well known that the GM profoundly display regulatory roles in different body systems [5].

The human GM contains approximately more than 100 trillion bacteria from more than 100 bacterial phyla and about 1000 different species. The GM encodes about 4,000,000 genes [6],[7], and the entire genome of the GM is called the “gut microbiome,” [8]. The GM also contains additional large numbers of some other microorganisms, including viruses, fungi, protozoa, and archaea [9]–[11]. Two phyla Firmicutes and Bacteroidetes constitute 70% of the total microbiota [12]. Other common bacteria in the human gut are proteobacteria, veromicrobiota, fusobacteria, cyanobacteria, actinobacteria, and spirochetes [13], which mostly reside in the colon. It is of noteworthy to point out that the microbial composition differs between different parts of the gut [14], as well as the lumen and the intestinal mucosa layer [15]. The human gut is extensively innervated, with neurons from the extrinsic and intrinsic plexuses [16],[17], which functionally have close relations to the GM.

Accumulating evidence suggests the GM plays a basic role in different physiological activities, such as the stimulation of the growth of microvilli, food digestion, maintenance of intestinal barrier integrity, improvement of the immune system, fermentation of dietary fibers, and inhibition of colonization of the digestive tract by harmful pathogens. Further, the GM plays a role in protecting against pathogenic organisms, metabolizing vital substances including (sterols, bile acids, and drugs), generating short-chain fatty acids (SCFAs), energy harvest and storage, synthesis of vitamins, neuronal activities, proliferation of neurons, brain functions, behaviors, social cognition, emotion, neurogenesis, neurotransmission, protection against oxidative stress, gastrointestinal motility, absorption of nutrients and the production of bioactive molecules [18]–[20]. Therefore, we should accept the opinion that, without the regulatory effects of GM on various systems, our body would be the target of many disorders [21]–[24].

### 1.1. Effects of postnatal factors on development of the GM

In the initial postnatal days, the GM is unstable and of low diversity [25]. By age 3, the GM composition stabilizes into an adult composition [26]. The GM is a highly dynamic system such that its density and composition can be affected by many postnatal factors including diet, lifestyle, treatment with drugs (particularly antibiotics), infections, mode of delivery at birth, stress, geography, genetic features, metabolism, immunity, hormones, age, and sex [27].

### 1.2. Eubiosis versus dysbiosis

The GM is shown to be involved in many physiological properties and body functions. Clinical reports support this hypothesis that a eubiotic GM composition is necessary for the maintenance of health. Eubiosis is characterized by the status in which beneficial species are predominant. They mainly belong to the two bacterial phyla, Firmicutes and Bacteroides, and a very small percentage of pathogenic species belonging to the phyla Proteobacteria [28]. On the other hand, decreased intestinal biodiversity or increased pathogenic bacteria, referred to as “dysbiosis”, leads to the development, prevalence, or prevention of numerous human disorders [29]–[31]. The majority of the GM alterations appear to be disease-specific, supporting the hypothesis that the GM can be used as a biomarker for the diagnosis of at least some disorders. A range of animal models in different investigation fields show that there is a correlation of more than 70% the between composition of the GM and disease parameters [32].

## 2. Purpose of using germ-free animal models

In many fields of host-microbe, interactions investigating germ-free (GF) animal models are appropriate experimental subjects. The use of GF animals facilitates the assessment of the role of the GM in all aspects of physiology, normal aging, and the nervous, digestive, immune systems, and metabolic function [33].

GF animal models provide biological model systems for studying either the complete absence of microbes, or colonized selected, and known microbes [34]. Experimental models using GF animals are valuable subjects to assess how the GM may affect host physiology [35]–[38]. In this regard, early studies show that the GM affects vascular remodeling in the intestine and increases vascular endothelial growth factor receptor 1 expression and vessel density [39]. Also, GM suppresses tonic Hedgehog (Hh) signaling in the small intestine, thus regulating intestinal barrier function. Hh pathway activity is mainly suppressed through Toll-like receptor (TLR2/TLR6) signaling in the intestinal epithelium, identifying intestinal epithelial neuropilin-1 (NRP1) as a microbiota-dependent Hh regulator that contributes to the stabilization of the intestinal epithelium barrier [40].

GF animal models are considerably used in evaluating the mechanistic understanding of microbe-induced changes in disease models [41]–[50], linking the GM dysbiosis with intestinal (irritable bowel syndrome, inflammatory bowel disease, etc.) and non-intestinal (metabolic syndrome, cancers, brain diseases, etc.) disorders. The GF animals can help us to elucidate the role of commensal microbiota in the development and function of the organism.

It is worth noting that, since those early models, advances in the knowledge of nutritional differences, intestinal morphology, intestinal epithelial properties, intestinal function, metabolic characteristics, and mucosal immunity [51],[52] have significantly developed GF animal models.

## 3. History of GF animal modeling

To test whether GF life of an animal host is possible, Nuttall and Thierfelder (1896) were the first to generate and manage to have them survive for 13 days [53]. They raised the first GF animals (guinea pigs), which were generated by aseptic caesarean section at the University of Berlin and kept them for 2 weeks [53]. However, because of the lack of knowledge concerning appropriate nutrition and adequate equipment, rearing and maintenance of healthy GF animals was a challenging task due to technological constraints until the mid-1900s when the first GF rat colony was established [54]. Nevertheless, GF research programs were developed independently at 3 different institutions and proved conclusively that life without microbes is possible, though not desirable. Systematic studies with GF animals started when, in the mid-twentieth century, a group headed by James Reyniers at the University of Notre Dame was the first to rear successive generations of GF rodents [55],[56]. Reyniers and his colleagues established academic organizations in the mid-1940s [55] and early 1950s devoted to understanding host–microbial interactions [57]. Almost simultaneously, Bengt Gustafsson at Lund University in Sweden also produced GF animals with a new isolation breeding system [58],[59]. Then, a third GF program started at Nagoya University under the leadership of Masasumi Miyakawa [60]. Pleasants (1959) developed the first GF mouse colony in the United States [61]. By the late 1950s, researchers were successful in rearing GF guinea pigs, mice, and chickens [56]. In the 1960s, life without GM was featured prominently in the medical, scientific, and public press, often reported as a compound of fact and fiction in the future. Then, after World War II, following the appearance of antibiotics, GF living became an interesting topic [62]. The concept of humans living in sterile worlds was realized as early as 1971, perhaps most notably with David Vetter, a patient with severe combined immunodeficiency who grew up in GF conditions as an infant and became known as “Bubble Boy”[62].

In industrial agriculture, GF animals are bred to make pathogen-free animals to help in veterinary work [62],[63]. Also, GF technology has been used to protect GF immunocompromised newborn [62],[64]–[67]. In spite of many applications of GF technology, it has not been widely implemented outside the laboratory yet. The main reason is that GF modeling is a labor-intensive technology that requires constant control to manage the state of cleanliness [68]–[70]. However, the existing methodology underlying the production of GF animals has remained essentially unchanged.

**Figure 1.**
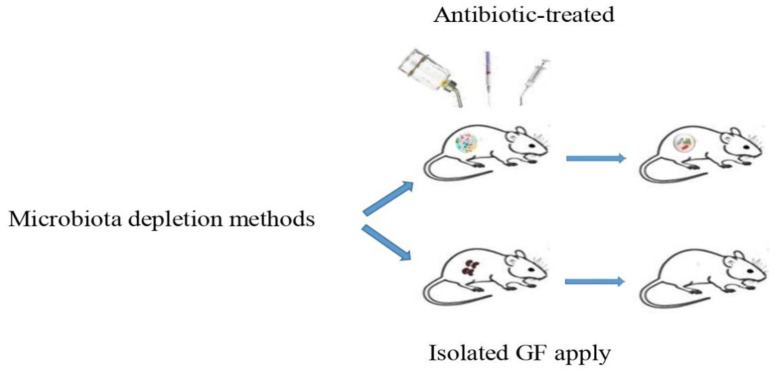
The processes involved in the generation of isolated GF and antibiotic-treated models.

With the advent of next generation sequencing and developments in microbial ecology, the use of gnotobiotic models is now a valuable resource for understanding host-microbe interactions in health and disease. Different methods are used in the study of GM, including isolated GF animals, antibiotic-treated animals, probiotic feeding, fecal microbiota transplantation, and mouse humanization. Methodologically, isolated GF and antibiotic-treated models are known as the main approaches by which exploring the effects of the microbiota on physiology and disease in mice is established (Figure 1). Both approaches have strengths and weaknesses [71],[72].

The colonization of GF animals with a minimal microbiome offers an attractive method to evaluate the etiology of disease-associated microbial changes [73]. In fact, the production of minimal microbiomes and their application in gnotobiotic models allow mechanistic studies of host-microbe interactions under controlled conditions [73].

### 3.1. Why mouse

Mice and rats are two of the most routine experiment rodents for GF animals; although, there are differences between the two rodents [74]. However, studies of host-microbe interactions are mostly focused on different species of laboratory mice. In addition to the characterization of the genotypes of mice [75], from the view of gene function, there is a very high similarity (99%) between genomes of mice and humans [76]. Furthermore, transgenic mice help us to induce genetic changes in an organism and evaluate the effects of these changes [77].

Different strains of mice can be used as GF models. For example, C57BL/ mice and 6 Swiss-Webster mice are used in GF models for study on type 2 diabetes mellitus and anxiety, respectively [32]. Moreover, the GM of GF mice can be “humanized” by transplantation of microbiota from feces of human patients or from animal models of diseases [78]–[87].

## 4. Isolated GF animal model

Isolated GF animals are born, bred, and raised for their whole lifetime in sterile isolators to prevent their exposure to microorganisms including bacteria, viruses, fungi, parasites, and protozoa, throughout its lifetime [88]–[90]. The key principle supporting the creation of isolated GF animals is the fact that the environment of uterine is sterile and colonization of the GI tract takes place after birth in normal humans and rodents [91]. Nevertheless, it is worth noting that researches on the potential for bacterial transfer across the placenta have also detected bacteria in placental tissue [92], umbilical cord blood [93], amniotic fluid [94]–[96], and fetal membranes [96],[97].

The production and transformation of isolated GF animals is difficult and expensive; however, environmental circumstances, isolator technology, and necessary equipment including feed, water, cages, and bedding have been improved, resulting in cost-effective systems [98]–[100].

To establish an initial isolated GF colony, newborns are carefully delivered by caesarean section to avoid contamination with microbes residing in the mother's vagina and skin [56],[59],[101]–[105]. The fetus is then delivered from the uterus in the isolator, and the neonate can be reared by a GF foster mother or artificially fed with formula milk [98]. Over the postnatal period of life, colonies are maintained in aseptic isolators in a GF unit where the food, water, and bedding are sterile, therefore eradicating the opportunity for postnatal colonization of their GI tract, allowing a direct comparison with the normal colonized gut of their counterparts.

**Figure 2.**
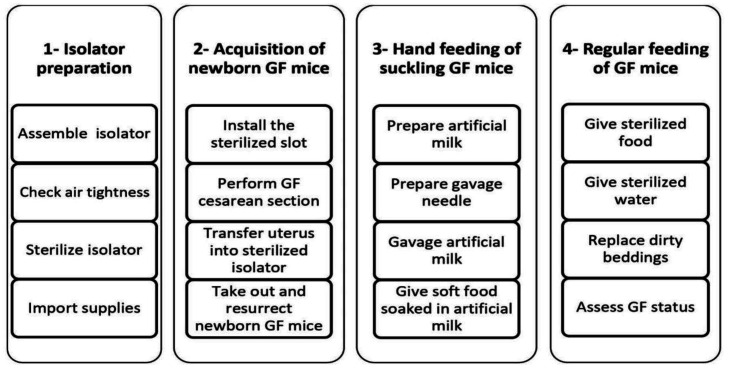
Overview of the procedures involved in establishing the isolated GF mice model.

Early isolated GF animals were housed in stainless steel isolators [59], then replaced by light, cheaper, more flexible, plastic polyvinyl chloride isolators [98],[106]. Figure 2 show the different stages of isolated GF animal production.

Isolated GF mice can also be reproduced by embryo transfer to axenic mice and then be kept in a GF isolator. The embryos are then removed from the uterus in the GF isolator and place on heating pads. These pups will be adopted by a foster mother [107]. A substitute method could be the transfer of an embryo at the 2-cell stage into a pseudo pregnant GF mother [102]–[104].

Although the creation of new strains of isolated GF animals requires that the fetus remains sterile in the uterus, however, the later descendents of isolated GF animals produce through a much simpler process. Isolated GF animals are mated and mothers can give birth naturally in the isolator without exposure to any microorganisms in the new litter [56],[59],[101]–[103]. The majority of commercially present isolated GF animals produced with this method can be transported in a sterile container to local GF facilities.

Methodologically, the use of first-generation isolated GF animals in experiments is imprudent because their mothers are not GF and some microbes or bacterial metabolites may be transferred from mother to the fetus through the placenta [52].

## 5. Antibiotic-treated animal model

Whereas isolated GF mouse models are mostly considered the gold standard for microbiota studies, the production and working with isolated GF animals, with strictly controlled identified microbes, is very difficult and expensive due to the use of isolators and rigid gnotobiotic working procedures.

As a result, depletion of the GM by antibiotic treatment, as a rapid, inexpensive, and accessible alternative has been considered for GF animals [108]. Actually, the antibiotic-induced short-term disruption of the GM in this model has been thought to be a less intrusive model to probe causality in microbiota-dependent effects [80],[109],[110]. As an alternative to isolated GF technology, treatment of mice with antibiotics is considered to be more practical and a potentially relevant alternative to GF mice [111].

### 5.1. Generation of antibiotic-treated GF animals

Broad-spectrum antibiotics are often used to deplete the existing microbiota, which can reduce the bacterial load by several orders of magnitude [112]–[114]. Researchers have used different regimens that differ in terms of antibiotic composition, dose, concentration, and duration of use. Common combinations of antibiotics usually do not surpass a mixture of five antibiotics at different doses, and may include penicillin, ampicillin, streptomycin, ciprofloxacin, vancomycin, neomycin, and metronidazole [85],[115]–[120]. All of these compounds broadly target Gram-negative, Gram-positive, and anaerobic bacteria [71]. The duration of antibiotic treatment is usually between 3 and 35 days, and the usual treatment time is 1 to 2 weeks [115],[119]–[121].

Table 1 summarizes different methods for the generation of antibiotic-treated animal model. In addition to antibiotics, some protocols include antifungals in the cocktail to avoid fungal overgrowth during treatment [122]–[124]. Sweeteners such as sugar, Kool-Aid, or Splenda may also be added to mask the bitterness and ensure that the animals drink the water containing antibiotics [112],[125],[126]. It is worth noting that there may be different findings based on the combination and dose of antibiotics, duration of treatment, the route of administration, and the age of the animals being tested.

Often, antibiotics are diluted in drinking water and animals are allowed to drink freely during the treatment period. Therefore, actual delivered doses may vary. Daily oral gavage can prevent dehydration and allow precise antibiotic dose delivery. Hence, this method is sometimes used alone or in combination with delivery in drinking water, although it is more labor intensive [122],[127]. It must be noted that, due to instability of antibiotics in solution, antibiotics mixtures must be freshly prepared daily.

**Table 1.** A summary of characteristics of studies on microbiota disrupted animals using the different regimens of antibiotics in the antibiotic-treated model.

Antibiotic administration	Antibiotics concentration	Duration	Additions	Animal	Sex	Age	Ref
Gavage	streptomycin (500 g/L)	2 days		C57BL/6 mouse	M	Young (8–12 W) Aged (18–20 months)	[171]
Drinking water	metronidazole (1 g/L) and ciprofloxacin (0.2 g/L)	2 days		C57BL/6JNarl mouse	M	5 W	[172]
Drinking water	neomycin (0.3 g), vancomycin (0.15 g) and aspartame (1.125 g) in 300 mL	3 days		C57Bl/6J mouse	M	8–12 W	[173]
Gavage	meropenem and vancomycin (50 mg/kg/day of each antibiotic)	3 days	Omeprazole (50 mg/kg/day)	Dahl rat	M	4 W	[174]
Gavage	bacitracin, streptomycin and neomycin (200 mg/kg /day each antibiotic)	3 days		C57BL/6 mouse	M	9–16 W	[175]
Gavage	Streptomycin, neomycin, and bacitracin (200 mg/kg of each antibiotic)	3 days		Swiss Webster mouse	M	12–15 W	[176]
Gavage	Neomycin, bacitracin, and streptomycin (200 mg/Kg of each antibiotic)	3 days		Wild-type C57BL/6J	M and F	8–12 W	[177]
Gavage	metronidazole, ampicillin, neomycin, gentamicin (1 mg/mL of each antibiotic), and vancomycin (0.5 mg/mL)	3 days		C57Bl/6 mouse	M And F	8–12 W	[178]
Drinking water	aspartame (1g/mL), vancomycin (0.1 mg/mL) and neomycin sulfate (0.2 mg/mL)	3 days		BALB/c mouse	F	6 W	[179]
Gavage	metronidazole, ampicillin (1 g/L of each antibiotic), vancomycin, and neomycin (0.5 g/L of each antibiotic)	3 days		C57BL/6N mouse	M And F	6–8 W	[180]
Gavage	Neomycin, ampicillin (500 mg of each antibiotic), vancomycin, and metronidazole (250 mg of each antibiotic)	3 days		C57BL/6 mouse	M and F	6 W	[181]
Gavage	streptomycin (5 g/L), vancomycin (0.25 g/L), ampicillin, and colistin (1 g/L of each antibiotic)	3 days		C57BL/6 and BALB/c mouse	F	7–12 W	[182]
Gavage	metronidazole, imipenem (90 mg/kg/day of each antibiotic), vancomycin (72 mg/kg/day), and ampicillin (180 mg/kg/day)	3 days		Sprague-Dawley rat	M	Young (~3 months) and aged (20~24 months)	[183]
Gavage	ampicillin (0.5 g/L), and ciprofloxacin (0.1 g/L)	5 days		C57BL/6 Jax mouse	F	6–10 W	[184]
Gavage	Ampicillin, neomycin, metronidazole sulfate (200 mg/kg of each antibiotic), and vancomycin (100 mg/kg)	5 days		C57BL/6 mouse	M	6–12 W	[185]
Drinking water	ampicillin (0.5 g/L)	5 days		C57BL/6J mouse	F	6–8 W	[186]
Gavage	Neomycin, ampicillin (100 mg/kg of each antibiotic), and metronidazole (50 mg/kg)	6 days		Wistar rat	M	6–8 W	[187]
Drinking water	ampicillin (1 g/L)	1 W		C57BL/6J mouse	M	7 W	[188]
Drinking water	clindamycin hydrochloride, ampicillin, ertapenem sodium, cefoperazone sodium salt, vancomycin hydrochloride, and neomycin sulfate (1 mg/ml of each antibiotic)	1 W		C57BL/6 mouse	F	6–8 W	[189]
Drinking water	Metronidazole, ampicillin, and neomycin (0.01 g/L Kg of each antibiotic)	1 W		C57BL/6J mouse	M	8–10 W	[190]
Drinking water	vancomycin, metronidazole (1 g/L of each antibiotic), and neomycin sulfate (0.5 g/L)	1 W		C57BL/6 mouse	M	6–8 W	[191]
Drinking water	ampicillin sodium, metronidazole, neomycin sulfate (1 mg/ml Kg of each antibiotic), and vancomycin hydrochloride (0.5 mg/ml)	1 W		Sprague Dawley rat	F	3 W	[192]
Drinking water	Ampicillin, clindamycin hydrochloride, cefoperazone sodium salt, neomycin sulfate, ertapenem sodium, and vancomycin hydrochloride. (1 mg/ml of each antibiotic)	1 W		C57BL/6J mouse	M and F	5–6 W	[193]
Gavage	nalidixic acid (200 mg/kg) OR clindamycin (67 mg/kg)	1 W		C57BL/6 Il10 -/- mouse	M And F	8–12 W	[194]
Drinking water	neomycin sulfate and streptomycin sulfate (100 mg/kg/L of each antibiotic)	1 W		Sprague-Dawley rat	M	8–10 W	[195]
Gavage	Bacitracin, neomycin (5 mg/ml of each antibiotic), and natamycin (2 mg/ml)	1 W		Wistar rat	M	16 W	[196]
Gavage OR Intraperitoneal injection	ampicillin, neomycin sulfate, metronidazole (1 g/ml of each antibiotic) and vancomycin (0.5 g/ml)	1 W	Sucrose (3%), Glucose (1%) OR Kool-Aid	SJL mouse	F	6 W	[197]
Gavage and Drinking water	**Gavage:** vancomycin (50 mg/kg), metronidazole neomycin and (100 mg/kg of each antibiotic) **Drinking water**: ampicillin (1 g/L)	1 W		wildtype Swiss Webster mouse	M and F	3–4 W	[198]
Gavage	vancomycin (100 mg/kg), ampicillin, metronidazole and neomycin (200 mg/kg of each antibiotic)	1 W		C57BL/6J mouse	M	3 and 8 W	[199]
Drinking water	vancomycin (0.5 g/kg), neomycin trisulfate, and metronidazole (1 g/kg of each antibiotic)	8 days		C57BL/6 mouse	F	7–8 W	[200]
Drinking water	Metronidazole, vancomycin (1 g/L of each antibiotic) and neomycin (0.5 g/L)	10 days	Sucrose (2%)	Swiss-Webster and C57BL/6 mouse	M and F	9 W	[201]
Gavage	ampicillin, metronidazole neomycin, gentamicin (1 mg/mL of each antibiotic) and vancomycin (0.5 mg/mL)	10 days		C57BL/6 mouse	F	6–8 W	[202]
Gavage	metronidazole (0.1 mg/g bodyweight), neomycin, ampicillin (0.26 mg/g bodyweight of each antibiotic), and vancomycin (0.13 mg/g bodyweight)	10 days		Ldlr−/− mouse	F	10–12 W	[203]
Drinking water	vancomycin, polymyxin B, metronidazole (1 g/L of each antibiotic), and cefotaxime (2 g/L)	10 days		C57BL/6 mouse	M		[204]
Drinking water	ampicillin, neomycin, metronidazole (1 g/L of each antibiotic) and vancomycin (500 mg/L)	10 days		C57BL/6 mouse	M	6–16 W	[205]
Gavage and Drinking water	**Gavage**: vancomycin (50 mg/kg), metronidazole, neomycin (100 mg/kg of each antibiotic), and amphotericin-B (1 mg/kg) **Drinking water:** ampicillin (1 mg/mL)	10 days		C57BL/6 mouse	M	12 W	[206]
Drinking water	amoxicillin-clavulanic acid (1 g/L)	1–2 W		C57BL/6 mouse	M	7–8 W	[207]
Gavage and Drinking water	**Gavage:** single dose of streptomycin (20 mg) **Drinking water**: ampicillin (1 g/L)	1–2 W		C57BL/6 mouse	M and F	6–12 W	[208]
Drinking water	ampicillin (1mg/ml) and neomycin (0.5 mg/mL)	1–2 W		C57BL/6 mouse	M And F	6–16 W	[209]
Drinking water	ampicillin, metronidazole, neomycin sulfate (1 g/L of each antibiotic) and vancomycin (0.5 g/L)	1–2 W		Wild-type C57BL/6 and Swiss mouse	F	6–10 W	[210]
Gavage	ampicillin (43.2 mg), meropenem (21.6 mg), vancomycin (6.48 mg), bacitracin and neomycin (108.0 mg of each antibiotic) in 4.5 mL of distilled water	1–2 W		C57BL/6N mouse	M	8–11 W	[152]
Gavage	ampicillin, metronidazole and neomycin sulfate (1 g/L of each antibiotic)	2 W		C57BL/6J mouse	M	8 W	[211]
Gavage	ampicillin, metronidazole and neomycin sulfate (1 g/L of each antibiotic)	2 W		NOD/ShiLtJ mouse	M	4 W	[212]
Drinking water	ampicillin, metronidazole, neomycin (0.2 g/L of each antibiotic), and vancomycin (0.1 g/L)	2 W		C57BL/6 mouse	M	6 W	[213]
Drinking water	ampicillin, neomycin, ciprofloxacin, metronidazole (1 g/L of each antibiotic) and vancomycin (0.5 g/L)	2 W	Grape flavored Kool-Aid (20 g/L)	CD45.1 OR CD45.2 C57BL/6 mouse	M	6–15 W	[214]
Gavage	ampicillin, neomycin, metronidazole, gentamicin (0.25 mg/day of each antibiotic), and vancomycin (0.125 mg/day)	2 W		C57BL/6J mouse	M	7 W	[215]
Gavage	ampicillin, neomycin, metronidazole (2.5 g/L of each antibiotic), and vancomycin (1.0 g/L)	2 W		C57BL/6 mouse	M	14–15 W	[216]
Drinking water	ampicillin, metronidazole, and neomycin sulfate (1 g/L of each antibiotic)	2 W		C57BL/6J mouse	M	8–10 W	[217]
Gavage	Vancomycin, ampicillin, neomycin, metronidazole (1 g/L of each antibiotic), erythromycin (10 mg/L), and gentamycin (100 mg/L)	2 W		C57BL/6J mouse	M		[218]
Gavage	Neomycin, ampicillin, metronidazole gentamicin (100 mg/kg of each antibiotic), and vancomycin (50 mg/kg)	2 W		Fischer rat	M	8 W	[219]
Gavage	neomycin, bacitracin (5 mg/mL of each antibiotic) and natamycin (2 mg/mL)	2 W		C57BL/6J mouse	M		[196]
Drinking water	ampicillin, metronidazole, and neomycin sulfate (1 g/L of each antibiotic)	2 W		Sprague-Dawley rat	M	6–8 W	[220]
Drinking water and Intraperitoneal injection	**Drinking water**: kanamycin (40 mg/L), metronidazole (21.5 mg/L), colistin (4.2 mg/L), and gentamicin (3.5 mg/L) **intraperitoneal injections:** vancomycin (200 µL of 0.5 mg/mL) only OR neomycin sulfate (500 mg/L), polymyxin B (1 g/L) in drinking water	2 W		BALB/c and C57BL/6 mouse	M	7–9 W	[221]
Drinking water	ampicillin (1 g/L), neomycin, vancomycin (0.5 g/L of each antibiotic), gentamycin (100 mg/L), and erythromycin (10 mg/L)	2 W		C57BL/6N mouse	M	4 W	[222]
Drinking water	neomycin, metronidazole, ampicillin (1 g/L of each antibiotic), and vancomycin (0.5 g/L)	2 W		C57BL/6 mouse and CXCR6-EGFP/+ mouse	M	8–10 W	[223]
Drinking water	ampicillin, metronidazole, neomycin (1 g/L), and vancomycin (0.5 g/L)	2 W		C57BL/6 mouse	M	6–14 W	[224]
Drinking water	ampicillin, metronidazole, neomycin (1 mg/mL of each antibiotic), and vancomycin (0.5 mg/mL)	2 W		C57BL/6J mouse	M	8–16 W	[225]
Drinking water	Ampicillin, metronidazole, neomycin (1 g/L of each antibiotic), and vancomycin (0.35 g/L)	2 W	Grape Kool-Aid (Kraft Foods) (25 g/L) OR Kool-Aid alone	C57BL/6J wild-type mouse	M and F	10–11 W	[226]
Drinking water	ampicillin, metronidazole, and neomycin sulfate (1 g/L of each antibiotic)	2 W		C57BL/6 mouse	M	8 W	[227]
Drinking water	ampicillin, neomycin metronidazole (1 g/L of each antibiotic), and vancomycin (0.5 g/L)	2 W	Glucose (1% w/v)	C57Bl/6 wild-type	M	8–12 W	[228]
Drinking water	vancomycin (0.5 g/L), metronidazole, neomycin and ampicillin (1 g/L of each antibiotic)	2 W		Wistar rat	M	48 W	[229]
Drinking water	ampicillin, metronidazole, and neomycin sulfate (1 g/L of each antibiotic)	2 W		C57BL/6 mouse	M	8 W	[230]
Drinking water	Metronidazole, vancomycin (1 g/L of each antibiotic), neomycin sulphate (500 mg/L), Alternatively clindamycin, and enrofloxacin (400 mg/L of each antibiotic)	2 W		C57BL/6 mouse	M		[231]
Gavage and Drinking water	**Gavage**: amphotericin B (0.1 g/L), vancomycin (5 g/L), neomycin, metronidazole (10 g/L), and **Drinking water**: ampicillin (1 g/L)	2 W	Omeprazole (50 mg/kg)	Sprague-Dawley rat	M and F	6–7 W	[232]
Gavage and Drinking water	**Gavage**: amphotericin-B (1 mg/kg) **Drinking water**: ampicillin (1 g/L)	2 weeks		BALB/c mouse	M	6–10 weeks	[122]
Gavage and Drinking water	**Gavage**: Vancomycin (250 mg), ampicillin, neomycin-sulfate, and metronidazole (500 mg of each antibiotic) in 500 mL water **Drinking water**: ampicillin (1 g/L)	2 W	Kool-Aid (10 g)	mouse	M and F	8–12 W	[233]
Drinking water	ampicillin, neomycin sulfate, metronidazole (1 g/L of each antibiotic), and vancomycin (500 mg/L)	2 W		C57BL6/J mouse	M and F	8–12 W	[234]
Gavage	ampicillin, neomycin, gentamicin, metronidazole (2 mg/mL of each antibiotic), and vancomycin (1 mg/mL)	2 W		C57BL/6 mouse	M	10–15 W	[235]
Drinking water	Ampicillin, neomycin, metronidazole (1g/L of each antibiotic), and vancomycin (0.5g/L)	2 W		C57BL/6 mouse	M	3–4 W	[236]
Drinking water	clindamycin (0.1 mg/mL), and streptomycin (5 mg/mL)	2 W		C57BL/6 mouse	F	6 W	[237]
Gavage and Drinking water	**Gavage**: ampicillin, metronidazole, neomycin and vancomycin (10 mg of each antibiotic per mouse per day) **Drinking water**: ampicillin, metronidazole, neomycin and (1 g/L of each antibiotic), and vancomycin (500 mg/L)	1–2 W		C57BL/6mouse	M	6 W	[238]
Gavage and Drinking water	**Gavage**: 3 days of amphotericinB (1 mg/kg) every 12 h + from day 3 **Drinking water**: ampicillin (1 g/L) **Oral Gavage**: every 12 h metronidazole, neomycin (100 mg/kg of each antibiotic), vancomycin (50 mg/kg), and amphotericin-B (1 mg/kg)	2–3 W		A/J strain mouse	M	7 W	[239]
Drinking water	ampicillin (1 mg/mL), and enrofloxacin (0.575 mg/mL)	2–4 W		C57BL/6 mouse	F	6–8 W	[240]
Drinking water	ampicillin, metronidazole, neomycin (1mg/mL of each antibiotic), and vancomycin (0.5 mg/mL)	2–4 W	Medi Drop Sucralose	C57BL/6 mouse	F	6–8 W	[240]
Drinking water	Ampicillin, colistin (1 mg/mL of each antibiotic), and streptomycin (5 mg/mL) OR imipenem alone (0.25 mg/mL) OR vancomycin alone (0.25 mg/mL) OR colistin alone (2.103 U/mL)	2–3 W		C57BL/6J and BALB/c mouse	F	7–14 W	[241]
Drinking water	Ampicillin, metronidazole, neomycin (1 g/L of each antibiotic), and vancomycin (0.5 g/L)	2–4 W		29X1SvJ mouse	M and F	6–14 W	[242]
Drinking water	Ampicillin, neomycin, metronidazole (1 g/L of each antibiotic), and vancomycin (0.5 g/L)	2–7 W		Swiss Webster mouse	M	4–10 W	[243]
Drinking water	vancomycin (100 mg/kg), metronidazole, neomycin sulfate, and ampicillin (200 mg/kg of each antibiotic)	3 W		C57BL/6J mouse	F	6 W	[244]
Gavage and Drinking water	kanamycin (4 mg/mL), colistin (8500 U/mL), gentamicin (0.35 mg/mL), vancomycin (0.45 mg/mL), and metronidazole (2.15 mg/mL)	3 W		C3H/HeJ and wild-type C3HeB/FeJ mouse	F	3 W	[245]
Drinking water	ampicillin (1 mg/mL), neomycin (10 mg/mL), and vancomycin (5 mg/mL)	3 W		C57BL/6 mouse	M		[246]
Drinking water	Metronidazole, ampicillin (1 mg/mL of each antibiotic), ciprofloxacin hydrochloride (0.2 mg/mL), vancomycin (5 mg/mL), and imipenem (0.25 mg/mL)	3 W		C57BL/6 mouse	M	4 and 10 W	[246]
Drinking water	ampicillin, kanamycin, neomycin, streptomycin, and (1 g/L of each antibiotic) AND/OR anti-fungal cocktail amphotericin (0.2 g/L), 5-fluorocytosine, and fluconazole (0.5 g/L of each antibiotic)	3 W		C57BL/6 mouse	M	6–8 W	[247]
Drinking water	Ampicillin, metronidazole, neomycin sulfate (1 g/L of each antibiotic), and vancomycin (0.5 g/L)	3 W		C57BL/6 mouse	M and F	10 and 12 W	[248]
Drinking water	vancomycin (0.5 g/L), kanamycin, ampicillin, and metronidazole (1 g/L of each antibiotic)	3 W		C57Bl/6 RORc-GFP mouse		8–9 W	[249]
Drinking water	Ampicillin, metronidazole, gentamicin, neomycin (0.5 mg/mL of each antibiotic) and vancomycin (0.25 mg/mL)	3 W		C57BL/6 mouse	M and F	6–10 W	[250]
Drinking water	Ampicillin, neomycin trisulfate, metronidazole (1 g/L of each antibiotic), and vancomycin (0.5 g/L)	3 W		C57BL/6 mouse	F	8–16 W	[251]
Drinking water	ampicillin, metronidazole, neomycin (1 mg/mL of each antibiotic) and vancomycin (0.5 mg/mL)	3 W		B10RIII mouse	M		[252]
Gavage	ampicillin, neomycin, metronidazole (1 g/L of each antibiotic), and vancomycin (0.5 g/L)	3 W		C57BL/6mouse	M	2 W	[253]
Gavage and Drinking water	**Gavage**: kanamycin (4 mg/mL), colistin (8500 U/mL), gentamicin (0.35 mg/mL), vancomycin (0.45 mg/mL), and metronidazole (2.15 mg/mL) **Drinking water**: the antibiotics were administered in the Drinking water at 50-fold dilution except for vancomycin, which was maintained at (0.5 mg/mL).	Gavage: 1 W followed by administration in water for 2 W		C57BL/6 or C57BL/6F mouse	M	2 W	[254]
Drinking water	vancomycin (0.5 g/L), metronidazole, ampicillin, and kanamycin (1 g/L of each antibiotic)	3 W		C57BL/6J mouse	M	6 W	[255]
Gavage	vancomycin (125 mg/kg), metronidazole, ampicillin, and kanamycin (250 mg/kg of each antibiotic)	3-4 W		C57BL/6 and BALB/c mouse	F	8–12 W	[256]
Drinking water	Vancomycin, metronidazole (0.5 g/L of each antibiotic), ampicillin, and neomycin (1 g/L of each antibiotic)	3–4 W	Sucrose (1% w/v)	C57BL/6 mouse	M	8–12 W	[256]
Drinking water	Metronidazol (0.5 g/mL), vancomycin, ampicillin, and streptomycin (1g/L of each antibiotic)	3-4 W		C57Bl/6 mouse	M	6–12 W	[257]
Drinking water	vancomycin (0.5 g/L), ampicillin, neomycin, and metronidazole (1 g/L of each antibiotic)	4 W	Polymixn B Sulfate (0.1 g/L)	C57BL/6 mouse	M		[258]
Drinking water	vancomycin (500 mg/L), ampicillin, metronidazole, and neomycin sulfate (1 g/L of each antibiotic)	4 W		C57BL/6 mouse	M	5 W	[259]
Drinking water	vancomycin (0.5 g/L) ampicillin, metronidazole, and neomycin (1 g/L of each antibiotic), together or separately	4 W	Grape Kool-Aid (20 g/L)	C57BL/6 mouse	F	3–4 W	[260]
Drinking water	vancomycin (500 mg/L), metronidazole, ampicillin, and neomycin sulfate (1 g/L of each antibiotic)	4 W		C57BL/6 mouse	M	6–7 W	[261]
Drinking water	vancomycin (0.5 g/L), metronidazole, ampicillin, and neomycin sulfate (1 g/L of each antibiotic)	4 W		C57BL/6 mouse	M	6–10 W	[262]
Drinking water	ampicillin, metronidazole, neomycin sulfate, (1 g/L of each antibiotic) and vancomycin hydrochloride (0.5 g/L)	4 W		C57BL/6 mouse	F	6–12 W	[263]
Drinking water	ampicillin, neomycin, metronidazole, and vancomycin (1 g/L of each antibiotic)	4 W		C57BL/6J mouse	F	3 W	[264]
Drinking water	neomycin (0.5 g/L), and ampicillin (1 g/L)	4 W		C57BL/6J mouse	M	10–13 W	[265]
Drinking water	vancomycin (500 mg/L), ampicillin, neomycin sulfate, and metronidazole (1 g/L of each antibiotic)	4 W		C57BL/6 mouse	M		[266]
Drinking water	vancomycin hydrochloride, metronidazole (0.5 g/L of each antibiotic), ampicillin, and neomycin sulfate (1 g/L of each antibiotic)	4 W		C57BL/6 mouse	M	16 W	[267]
Drinking water	vancomycin (0.5 g/L), ampicillin, and polymyxin (0.1 g/L of each antibiotic)	4 W		BALB/c, and C57BL/6 mouse	M		[268]
Drinking water	vancomycin (500 mg/L), neomycin sulfate, ampicillin, and metronidazole (1 g/L of each antibiotic)	4 W		C57BL/6 mouse	M	6 W	[269]
Drinking water	neomycin sulfate (0.5 mg/mL), ampicillin, metronidazole, and vancomycin (1 mg/mL of each antibiotic)	4 W		C57BL/6 mouse	M	6–8 W	[270]
Drinking water	vancomycin (0.5 g/L), ampicillin, metronidazole, and neomycin (1 g/L of each antibiotic)	4 W		C57BL/6J mouse)	M	4 W	[271]
Drinking water	vancomycin (500 mg/L), neomycin sulfate, ampicillin, and metronidazole (1 g/L of each antibiotic)	4 W		C57B/6 mouse	M		[272]
Drinking water	vancomycin (500 mg/L), neomycin sulfate, ampicillin, and metronidazole (1 g/L of each antibiotic)	4 W		C57BL/6 mouse	M		[273]
Drinking water	vancomycin (0.50 mg/mL), neomycin sulfate, ampicillin, and metronidazole (1 mg/mL of each antibiotic)	4 W		C57BL/6 mouse	M		[274]
Drinking water	Cefoxitin, metronidazole, gentamycin sulfate, and vancomycin (1 mg/mL of each antibiotic)	4 W		C57BL/6J mouse	F And M	4–6 W	[275]
Drinking water	vancomycin (500 mg/L), neomycin sulfate, ampicillin and metronidazole (1 g/L of each antibiotic)	4 W		C57BL/6J mouse	M	4 W	[276]
Drinking water	vancomycin (0.5 mg/mL), metronidazole, ampicillin and neomycin (1 mg/ml of each antibiotic) OR vancomycin (0.5mg/mL) alone	4 W		BALB/c mouse	F	8 W	[277]
Drinking water	Vancomycin (0.5 mg/mL), metronidazole, ampicillin, and neomycin (1 mg/mL of each antibiotic)	4 W		Apc ^Min/+^ mouse	M	6 W	[278]
Drinking water	vancomycin, ampicillin, and neomycin	4 W		C57BL/6J mouse	M	4 W	[279]
Drinking water	Vancomycin (0.5 mg/mL), ampicillin, streptomycin, and neomycin sulfate (1 mg/mL of each antibiotic)	4-5 W		C57BL/6 mouse	M	8–12 W	[280]
Drinking water	Ampicillin, metronidazole, streptomycin, and vancomycin (1g/L of each antibiotic)	4-6 W		C57BL/6 mouse	M	6–8 W	[281]
Drinking water	vancomycin (500 mg/L), ampicillin, metronidazole, and neomycin trisulfate (1 g/L of each antibiotic)	4-8 weeks		C57BL/6 mouse	F	3 W	[282]
Drinking water	vancomycin (0.045 mg/mL), kanamycin (0.4 mg/mL), colistin (850 U/mL), gentamicin (0.035 mg/mL), and metronidazole (0.215 mg/mL)	5 W		Sprague-Dawley rat	M	6–8 W	[283]
Gavage and Drinking water	**Gavage**: metronidazol (0.4 mg), streptomycin (2 mg), and colistin (0.3 mg) **Drinking water**: vancomycin (0.25 mg/ml)	5 W	Amphotericin-B (20 µg)	BALB/c or C57BL/6 mouse	F	7–10 W	[284]
Gavage and Drinking water	**Gavage**: ampicillin, metronidazole, neomycin, and vancomycin (10 mg/day) for 5 days **Drinking water**: vancomycin (0.5 g/L), ampicillin, metronidazole, and neomycin (1 g/L of each antibiotic)	5–6 W		C57BL/6 mouse	M	4 W	[285]
Drinking water	vancomycin (0.5 g/L), metronidazole, ampicillin, and neomycin (1 g/L of each antibiotic)	6 W	Sucrose	NZBWF1 mouse	F	26 W	[286]
Drinking water	ampicillin, colistin (1 g/L of each antibiotic), and streptomycin (5 g/L)	6–7 W	Sucrose (2.5% w/v)	*Rorc*-GFP mouse	M	6 W	[287]
Drinking water	ciprofloxacin (200 mg/L), imipenem, cilastatin (250 mg/L of each antibiotic), vancomycin (500 mg/L), ampicillin, and metronidazole (1 g/L of each antibiotic)	7 W		C57BL/6J mouse	M	6–8 W	[288]
Drinking water	Metronidazole and vancomycin (0.5–1.0 g/L)	10 W		C57BL/6J mouse	M		[289]
Drinking water	vancomycin, kanamycin, metronidazole, and ampicillin (1 g/L of each antibiotic)			C57Bl/6 and outbred Swiss Webster mouse	F	8–9 W	[290]
Gavage and Drinking water	**Gavage**: streptomycin (100 mg) **Drinking water**: vancomycin (0.5 g/L), ampicillin, metronidazole, and neomycin (1 g/L of each antibiotic)	More than 1 W	Sucrose (1%)	C57BL/6J mouse	M And F	6–8 W	[291]

M: male, F: female, W: week

## 6. Validation of bacterial depletion

An important aspect of working with GF animals is to confirm that the animals are without any microbes. Three screening methods are commonly used to access GF status. These include anaerobic/aerobic liquid culture, Gram-stain, and polymerase chain reaction (PCR) using universal and specific 16S rRNA bacterial primers [36].

In fact, the fastest way to confirm the presence of any microbes is culture-based methods by evaluating colony-forming units (CFUs) from fecal samples placed in aerobic and/or anaerobic conditions on non-selective media, or alternatively by making Gram-stained fecal smears and assessing them under a light microscope [71]. The technician swabs the cages and, using bacterial culture methods, analyzes stool samples to confirm that the GF unit is truly sterile [104],[128]. These methods ensure that the GF mice do not have a contact with any bacteria in the gut and on any other surface. Furthermore, they only consider cultivable microbes.

Recent studies show that, in practice, bacterial culture and Gram-stain are sufficient to screen for GF status because both have high sensitivity and specificity, while PCR shows high specificity but less sensitivity [129]. However, each technique has limitations. Cultivating GM outside the intestine requires an anaerobic environment, such as an anaerobic chamber, which is costly. Hence, the GF status of mice must be frequently monitored by fecal sample culturing for aerobic/anaerobic bacteria and fungi. Here, performing molecular techniques, such as PCR amplification, is a good alternative for bacteria that cannot be cultured [52],[102]. Quantitative PCR of the gene encoding 16S rRNA also allows for culture-independent evaluation of the bacterial load of the gastrointestinal tract [71]. However, it should be noted that bacterial 16S rRNA gene contamination in the breeding diet can occur, thus, PCR-based control should not be considered as the only sterility test for PCR-based sterility controls.

## 7. Advantages of isolated GF animal model

Isolated GF animals seem to be the best controlled models for microbial transplantation, and this model has been subject of the most experimental research on the GM so far. As previously described, fecal microbiota transplantation is a method in which selected bacteria can be transfered from a donor subject to a recipient one. Because isolated GF animals are free of microorganisms, they are a good model system for the response to bacterial introduction; indicating that they are suitable for studying the effects of microbes on host development and function. On the other hand, in vivo experimental models show a reduction or absence of several inflammatory and complex diseases in isolated GF animals; suggesting that the GM is associated with the development of these diseases [130]. Therefore, it is not surprising that isolated GF mice live longer than normally colonized control animals [131]–[133]. It is probably due to the absence of pathological infections. Therefore, this animal model provides conditions through which the positive and negative role of the GM on lifespan can be evaluated.

## 8. Disadvantages of isolated GF animal model

Despite the many advantages that the isolated GF model has, some disadvantages can limit their use. First of all, since these animals are never exposed to microorganisms, they display impaired physiology and immune development from birth. Technically, the production and maintenance of isolated GF animals need particular facilities, and the cost, labor, and skills necessary to preserve them can make these models inaccessible to many investigators [71].

Isolated GF mice should be regularly monitored for contamination using a combination of culture, microscopy, serology, gross morphology, and sequence-based diagnostic techniques [34],[129] and this limits the number of different genotypes that can be studied. Additionally, keeping animals in isolators may make some studies (e.g., behavioral testing or pathogen infections) impractical or challenging [71].

Growing evidence shows that isolated GF animals have some biochemical and physiological abnormalities such as altered immune systems [43], mild chronic diarrhea [134] and impaired metabolism [135], and reduced reproduction [136]. Particularly, immune system is known to be primed by the GM in early life [42],[137]–[140]. The immune response to fecal microbiota transplantation in isolated GF mice, which have never previously encountered the bacteria, must be expected to be important for at least some disease models [108].

## 9. Advantages of antibiotic-treated animal model

The advantage of antibiotic-treated over isolated GF studies is the timing of the GM changes or reductions and translatability to humans [141]. Treatment with broad-spectrum antibiotics is commonly used to eliminate the GM in mice and can be easily applied to any mouse genotype or condition [71]. Because of differences in their action mechanism, antibiotics are able to selectively exhaust different types of microbes.

**Figure 3.**
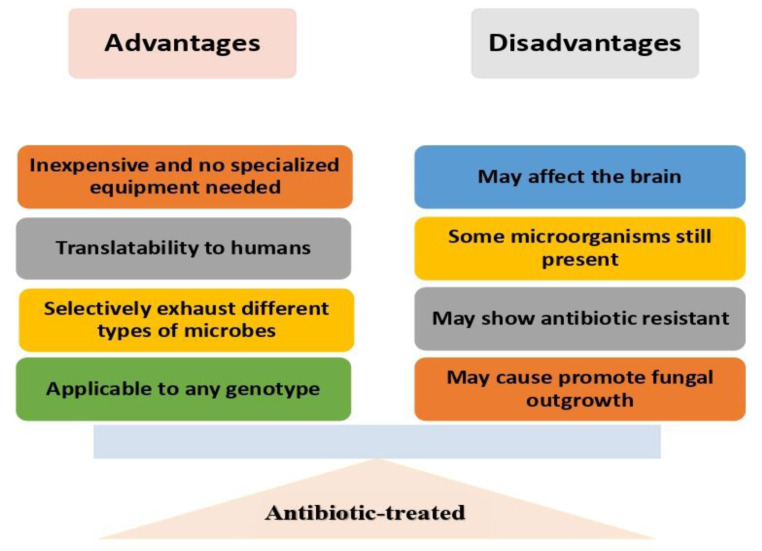
Illustration of advantages and disadvantages of antibiotic-treated animal model.

Individual antibiotics can be used to alter the GM composition in order to identify bacterial classes associated with different phenotypes [142],[143]. A cocktail of different classes of antibiotics can be used to broadly deplete the GM [71]. Antibiotics also have the advantage of allowing the examination of the consequences of intestinal microbial depletion at different stages of life [144]. In fact, by targeting different groups of bacteria through different classes of antibiotics, it is possible to develop hypotheses about which bacteria are responsible for disease manifestations. For instance, while both clindamycin and metronidazole target anaerobes, polymyxin B specifically targets Gram-negative bacteria and vancomycin is only effective against Gram-positive bacteria. [142],[145]. It is also possible to transfer host phenotypes with normal GM to antibiotic-treated animals through fecal microbiota transplantation, however problems related to reproducibility and antibiotic resistance genes must be considered [108] (Figure 3).

## 10. Disadvantages of antibiotic-treated animal model

Antibiotic-induced dysbiosis presents several challenges, especially when used for fecal microbiota transplantation studies. Although a broad-spectrum antibiotic approach significantly reduces most bacterial species, bacteria will still remain in the gut, as demonstrated by denaturing gradient gel electrophoresis [111] or cultivation [146]. It is difficult to precisely control the effect of an antibiotic administration in terms of species are completely eradicated and which species are only reduced, and the residual microbiota from antibiotic-treated mice may also influence colonization over time [108]. Because the immune system is primed by the GM at early postnatal age [42],[137]–[139], exposure to microbes prior to elimination with antibiotics can have long-term effects on the physiology of the host. An important potential drawback of eliminating microbiota with antibiotics using broad-spectrum antibiotics can cause the evolution or development of antibiotic-resistant bacteria and the selection and overgrowth of resistant bacterial species [147]–[149]. It may play a significant dominant role in the microbial profile after recolonization [146] or may be detrimental to animal health.

Although oral administration of antibiotics decreases the GM, other microbial communities, for instance the skin and lung microbiota, are not ever directly affected. It depends on the pharmacokinetics of the antibiotic substance and may also developmentally affect the immune system [150],[151]. Also, if antibiotics are administered through drinking water, the possible disadvantages of antibiotic-induced intestinal dysbiosis could be the systemic or even central effects of the antibiotics themselves as well as changes in consumption [152]. Antibiotics may even directly affect the brain. There is evidence that they can modulate the vagus nerve [153] and the enteric nervous system [154]. Mounting evidence indicates that bacteriophages, fungi, and eukaryotic viruses, which are not directly targeted by antibacterial antibiotics, cannot be discounted in GM homeostasis and immune priming [155],[156]. In addition, antibiotic therapy can allow the overgrowth of common fungal species, possibly confusing results because these organisms can modify immune function [157],[158] (Figure 3).

## 11. Effects of dysbiosis on other organs

Microbiota disruption affects the anatomy and function of various organs, such as the liver and gastrointestinal tract [159],[160]. One of the most obvious anatomical changes is the enlargement of the cecum, due to mucus and undigested fiber accumulation, which is observed in both isolated GF and antibiotic-treated mice [34],[122]. In addition, GF mice have elongated villus structures, reduced villus width, and weakened capillary networks in small intestinal villi [133],[161],[162]. The immune cell populations are also influenced by antibiotic treatment [163]–[165]. Oral antibiotic regimens have been shown to reduce cultivable bacteria in the respiratory system tract [114],[125],[166] and vagina of mice [167] with no effect on skin bacterial communities [168].

## 12. Conclusion

The importance of the GM in physiology of almost all body organs has encouraged research in this field. However, there are numerous problems in research strategies in the human GM. Therefore, animal models take a major role in many aspects of GM investigations. There are two very often used animal models: the isolated GF and antibiotic-treated models. Each of these models display advantages and disadvantages. Whereas both models are currently used by researchers, there are numerous differences between the two models such that, it is suggested that they should be viewed as distinct models for the GM manipulation. Consequently, it is very important to apply appropriate models when working on the GM. On one hand, because isolated GF animals are not exposed to bacteria from conception onwards, their use for experimental questions about the impact of altered microbiota composition in postnatal life may be limited [169]. On the other hand, given the experimental variables and several side effects of antibiotic- treated protocols, it is increasingly evident that the interpretation of data collected from experiments on microbiota disrupted by antibiotics should be approached with caution. A possible approach to circumvent the uncontrolled situation of antibiotic-treated animals and the effect of GF early in life is to use the generation of GF parents and use succeeding generations [108].

Accordingly, it is believed that findings obtained from GF animal models should be used with caution to develop strategies for the disease treatment and/or prevention [170]. Taken together, laboratory animals are currently the major models for the study of the GM, and each model has its own limitations; nevertheless, reproducibility must always be emphasized as an undisputed essential feature of the system.

## References

[1] Tillisch K (2014). The effects of gut microbiota on CNS function in humans. Gut Microbes.

[2] Mackowiak PA (2013). Recycling metchnikoff: probiotics, the intestinal microbiome and the quest for long life. Front Public Health.

[3] Al-Asmakh M, Hedin L (2015). Microbiota and the control of blood-tissue barriers. Tissue Barriers.

[4] McKenney PT, Pamer EG (2015). From Hype to Hope: The gut microbiota in enteric infectious disease. Cell.

[5] Novotny M, Klimova B, Valis M (2019). Microbiome and cognitive impairment: can any diets influence learning processes in a positive way?. Front Aging Neurosci.

[6] Rup L (2012). The human microbiome project.

[7] Hu X, Wang T, Jin F (2016). Alzheimer's disease and gut microbiota. Sci China Life Sci.

[8] Petschow B, Doré J, Hibberd P (2013). Probiotics, prebiotics, and the host microbiome: the science of translation. Ann N Y Acad Sci.

[9] Qin J, Li R, Raes J (2010). A human gut microbial gene catalogue established by metagenomic sequencing. Nature.

[10] Ashida H, Ogawa M, Kim M (2012). Bacteria and host interactions in the gut epithelial barrier. Nat Chem Biol.

[11] Moschen AR, Wieser V, Tilg H (2012). Dietary factors: major regulators of the gut's microbiota. Gut Liver.

[12] Mariat D, Firmesse O, Levenez F (2009). The Firmicutes/Bacteroidetes ratio of the human microbiota changes with age. BMC Microbiol.

[13] Backhed F, Ley RE, Sonnenburg JL (2005). Host-bacterial mutualism in the human intestine. Science.

[14] Frank DN, St Amand AL, Feldman RA (2007). Molecular-phylogenetic characterization of microbial community imbalances in human inflammatory bowel diseases. Proc Natl Acad Sci U S A.

[15] Swidsinski A, Loening-Baucke V, Lochs H (2005). Spatial organization of bacterial flora in normal and inflamed intestine: a fluorescence in situ hybridization study in mice. World J Gastroenterol.

[16] Furness JB (2006). The organisation of the autonomic nervous system: peripheral connections. Auton Neurosci.

[17] Blackshaw LA, Brookes S, Grundy D (2007). Sensory transmission in the gastrointestinal tract. Neurogastroenterol Motil.

[18] Alkasir R, Li J, Li X (2017). Human gut microbiota: the links with dementia development. Protein Cell.

[19] Quigley EMM (2017). Microbiota-brain-gut axis and neurodegenerative diseases. Curr Neurol Neurosci Rep.

[20] Felice VD, O'Mahony SM (2017). The microbiome and disorders of the central nervous system. Pharmacol Biochem Behav.

[21] Ding HT, Taur Y, Walkup JT (2017). Gut microbiota and autism: key concepts and findings. J Autism Dev Disord.

[22] Lubomski M, Tan AH, Lim SY (2019). Parkinson's disease and the gastrointestinal microbiome. J Neurol.

[23] Painold A, Morkl S (2019). A step ahead: Exploring the gut microbiota in inpatients with bipolar disorder during a depressive episode.

[24] Zhuang ZQ, Shen LL, Li WW (2018). Gut microbiota is altered in patients with alzheimer's disease. J Alzheimers Dis.

[25] Arrieta MC, Stiemsma LT, Amenyogbe N (2014). The intestinal microbiome in early life: health and disease. Front Immunol.

[26] Voreades N, Kozil A, Weir TL (2014). Diet and the development of the human intestinal microbiome. Front Microbiol.

[27] Foster JA, McVey Neufeld KA (2013). Gut-brain axis: how the microbiome influences anxiety and depression. Trends Neurosci.

[28] Zhang YJ, Li S, Gan RY (2015). Impacts of gut bacteria on human health and diseases. Int J Mol Sci.

[29] Lin CS, Chang CJ, Lu CC (2014). Impact of the gut microbiota, prebiotics, and probiotics on human health and disease. Biomed J.

[30] Norman JM, Handley SA, Virgin HW (2014). Kingdom-agnostic metagenomics and the importance of complete characterization of enteric microbial communities. Gastroenterology.

[31] Palm NW, de Zoete MR, Flavell RA (2015). Immune-microbiota interactions in health and disease. Clin Immunol.

[32] Hansen AK, Hansen CHF, Krych L (2014). Impact of the gut microbiota on rodent models of human disease. World J Gastroenterol.

[33] Grenham S, Clarke G, Cryan JF (2011). Brain–gut–microbe communication in health and disease. Front Physiol.

[34] Nicklas W, Keubler L, Bleich A (2015). Maintaining and monitoring the defined microbiota status of gnotobiotic rodents. ILAR J.

[35] Grover M, Kashyap PC (2014). Germ-free mice as a model to study effect of gut microbiota on host physiology. Neurogastroenterol Motil.

[36] Bhattarai Y, Kashyap PC, Proetzel G., Wiles M.V. (2016). Germ-free mice model for studying host–microbial interactions. Mouse Models for Drug Discovery.

[37] Fiebiger U, Bereswill S, Heimesaat MM (2016). Dissecting the interplay between intestinal microbiota and host immunity in health and disease: lessons learned from germfree and gnotobiotic animal models. Eur J Microbiol Immunol.

[38] Kubelkova K, Benuchova M, Kozakova H (2016). Gnotobiotic mouse model's contribution to understanding host–pathogen interactions. Cell Mol Life Sci.

[39] Reinhardt C, Bergentall M, Greiner TU (2012). Tissue factor and PAR1 promote microbiota-induced intestinal vascular remodelling. Nature.

[40] Pontarollo G, Kollar B, Mann A (2023). Commensal bacteria weaken the intestinal barrier by suppressing epithelial neuropilin-1 and Hedgehog signaling. Nat Metab.

[41] Bäckhed F, Ding H, Wang T (2004). The gut microbiota as an environmental factor that regulates fat storage. Proc Natl Acad Sci.

[42] Hansen CHF, Nielsen DS, Kverka M (2012). Patterns of early gut colonization shape future immune responses of the host. PloS one.

[43] Mazmanian SK, Liu CH, Tzianabos AO (2005). An immunomodulatory molecule of symbiotic bacteria directs maturation of the host immune system. Cell.

[44] Neufeld K-AM, Kang N, Bienenstock J (2011). Effects of intestinal microbiota on anxiety-like behavior. Commun Integr Biol.

[45] Olszak T, An D, Zeissig S (2012). Microbial exposure during early life has persistent effects on natural killer T cell function. Science.

[46] Schéle E, Grahnemo L, Anesten F (2013). The gut microbiota reduces leptin sensitivity and the expression of the obesity-suppressing neuropeptides proglucagon (Gcg) and brain-derived neurotrophic factor (Bdnf) in the central nervous system. Endocrinology.

[47] Sudo N, Chida Y, Aiba Y (2004). Postnatal microbial colonization programs the hypothalamic–pituitary–adrenal system for stress response in mice. J Physiol.

[48] Bleich A, Mähler M (2005). Environment as a critical factor for the pathogenesis and outcome of gastrointestinal disease: experimental and human inflammatory bowel disease and helicobacter-induced gastritis. Pathobiology.

[49] Sellon RK, Tonkonogy S, Schultz M (1998). Resident enteric bacteria are necessary for development of spontaneous colitis and immune system activation in interleukin-10-deficient mice. Infection and immunity.

[50] Song F, Ito K, Denning TL (1999). Expression of the neutrophil chemokine KC in the colon of mice with enterocolitis and by intestinal epithelial cell lines: effects of flora and proinflammatory cytokines. J Immunol.

[51] Wostmann BS (1981). The germfree animal in nutritional studies. Annu Rev Nutr.

[52] Al-Asmakh M, Zadjali F (2015). Use of germ-free animal models in microbiota-related research. J Microbiol Biotechnol.

[53] Nuttall GH, Thierfelder H (1896). Thierisches leben ohne bakterien im verdauungskanal. Biol Chem.

[54] Gustafsson B (1946). Germ-free rearing of rats. Cells Tissues Organs.

[55] Reyniers JA (1946). Rearing germfree albino rats. Lobund reports.

[56] Reyniers JA (1959). The pure-culture concept and gnotobiotics. Ann N Y Acad Sci.

[57] Bruckner G (1997). How it started-and what is mas?. Institute Microbiol Biochem.

[58] Gustafsson B, Kahlson G, Rosengren E (1957). Biogenesis of histamine studied by its distribution and urinary excretion in germ free reared and not germ free rats fed a histamine free diet. Acta Physiol Scand.

[59] Gustafsson BE (1959). Lightweight stainless steel systems for rearing germfree animals. Ann N Y Acad Sci.

[60] Miyakawa M (1959). The miyakawa remote-control germfree rearing unit. Ann N Y Acad Sci.

[61] Pleasants JR (1959). Rearing germfree cesarean-born rats, mice, and rabbits through weaning. Ann N Y Acad Sci.

[62] Kirk RG (2012). “Life in a germ-free world”: isolating life from the laboratory animal to the bubble boy. Bull Hist Med.

[63] Betts A, Trexler P (1969). Development and possible uses for gnotobiotic farm animals. The Vet Rec.

[64] Barnes R, Tuffrey M, Cook R (1968). A “germfree” human isolator. Lancet.

[65] Barnes R, Bentovim A, Hensman S (1969). Care and observation of a germ-free neonate. Arch Dis Child.

[66] Barnes R, Fairweather D, Holliday J (1969). A germfree infant. Lancet.

[67] Lawrence RJ (1985). David the ‘bubble boy’ and the boundaries of the human. JAMA.

[68] Wullaert A, Lamkanfi M, McCoy KD (2018). Defining the impact of host genotypes on microbiota composition requires meticulous control of experimental variables. Immunity.

[69] Macpherson A, McCoy K (2015). Standardised animal models of host microbial mutualism. Mucosal Immunol.

[70] Dremova O, Mimmler M, Paeslack N (2023). Sterility testing of germ-free mouse colonies. Front Immunol.

[71] Kennedy EA, King KY, Baldridge MT (2018). Mouse microbiota models: comparing germ-free mice and antibiotics treatment as tools for modifying gut bacteria. Front Physiol.

[72] Bayer F, Ascher S, Pontarollo G (2019). Antibiotic treatment protocols and germ-free mouse models in vascular research. Front Immunol.

[73] Bolsega S, Bleich A, Basic M (2021). Synthetic microbiomes on the rise—application in deciphering the role of microbes in host health and disease. Nutrients.

[74] Qv L, Yang Z, Yao M (2020). Methods for establishment and maintenance of germ-free rat models. Front Microbiol.

[75] Wiles MV, Taft RA, Proetzel G., Wiles M.V. (2010). The sophisticated mouse: protecting a precious reagent. Mouse Models for Drug Discovery: Methods and Protocols.

[76] Mouse Genome Sequencing Consortium (2002). Initial sequencing and comparative analysis of the mouse genome. Nature.

[77] Doetschman T, Gregg RG, Maeda N (1987). Targetted correction of a mutant HPRT gene in mouse embryonic stem cells. Nature.

[78] Turnbaugh PJ, Ley RE, Mahowald MA (2006). An obesity-associated gut microbiome with increased capacity for energy harvest. Nature.

[79] Turnbaugh PJ, Ridaura VK, Faith JJ (2009). The effect of diet on the human gut microbiome: a metagenomic analysis in humanized gnotobiotic mice. Sci Transl Med.

[80] Bercik P, Denou E, Collins J (2011). The intestinal microbiota affect central levels of brain-derived neurotropic factor and behavior in mice. Gastroenterology.

[81] Borody TJ, Khoruts A (2012). Fecal microbiota transplantation and emerging applications. Nat Rev Gastroenterol Hepatol.

[82] Cryan JF, Dinan TG (2012). Mind-altering microorganisms: the impact of the gut microbiota on brain and behaviour. Nat Rev Neurosci.

[83] Aroniadis OC, Brandt LJ (2013). Fecal microbiota transplantation: past, present and future. Curr Opin Gastroenterol.

[84] Ridaura VK, Faith JJ, Rey FE (2013). Gut microbiota from twins discordant for obesity modulate metabolism in mice. Science.

[85] Suez J, Korem T, Zeevi D (2014). Artificial sweeteners induce glucose intolerance by altering the gut microbiota. Nature.

[86] Thaiss CA, Zeevi D, Levy M (2014). Transkingdom control of microbiota diurnal oscillations promotes metabolic homeostasis. Cell.

[87] Leone V, Gibbons SM, Martinez K (2015). Effects of diurnal variation of gut microbes and high-fat feeding on host circadian clock function and metabolism. Cell Host Microbe.

[88] Wostmann BS (2020). Germfree and gnotobiotic animal models: background and applications.

[89] Yi P, Li L (2012). The germfree murine animal: an important animal model for research on the relationship between gut microbiota and the host. Vet Microbiol.

[90] Gonzalez-Arancibia C, Urrutia-Pinones J, Illanes-Gonzalez J (2019). Do your gut microbes affect your brain dopamine?. Psychopharmacology.

[91] Stinson LF, Payne MS, Keelan JA (2017). Planting the seed: origins, composition, and postnatal health significance of the fetal gastrointestinal microbiota. Crit Rev Microbiol.

[92] Aagaard K, Ma J, Antony KM (2014). The placenta harbors a unique microbiome. Sci Transl Med.

[93] Jiménez E, Fernández L, Marín ML (2005). Isolation of commensal bacteria from umbilical cord blood of healthy neonates born by cesarean section. Curr Microbiol.

[94] Bearfield C, Davenport ES, Sivapathasundaram V (2002). Possible association between amniotic fluid micro-organism infection and microflora in the mouth. BJOG.

[95] Jiménez E, Marín ML, Martín R (2008). Is meconium from healthy newborns actually sterile?. Res Microbiol.

[96] Rautava S, Collado MC, Salminen S (2012). Probiotics modulate host-microbe interaction in the placenta and fetal gut: a randomized, double-blind, placebo-controlled trial. Neonatology.

[97] Steel JH, Malatos S, Kennea N (2005). Bacteria and inflammatory cells in fetal membranes do not always cause preterm labor. Pediatr Res.

[98] Arvidsson C, Hallén A, Bäckhed F (2012). Generating and analyzing germ-free mice. Curr Protoc Mouse Biol.

[99] McCoy KD, Geuking MB, Ronchi F (2017). Gut microbiome standardization in control and experimental mice. Curr Protoc Immunol.

[100] Walter J, Hornef MW (2021). A philosophical perspective on the prenatal in utero microbiome debate. Microbiome.

[101] Macpherson AJ, Harris NL (2004). Interactions between commensal intestinal bacteria and the immune system. Nat Rev Immunol.

[102] Smith K, McCoy KD, Macpherson AJ (2007). Use of axenic animals in studying the adaptation of mammals to their commensal intestinal microbiota. Semin Immunol.

[103] Faith JJ, Rey FE, O'donnell D (2010). Creating and characterizing communities of human gut microbes in gnotobiotic mice. ISME J.

[104] Bibiloni R (2012). Rodent models to study the relationships between mammals and their bacterial inhabitants. Gut Microbes.

[105] Stilling RM, Dinan TG, Cryan JF (2014). Microbial genes, brain & behaviour–epigenetic regulation of the gut–brain axis. Genes Brain Behav.

[106] Basic M, Bleich A (2019). Gnotobiotics: Past, present and future. Lab Anim.

[107] Bhattarai Y, Kashyap PC, Proetzel G., Wiles M.V. (2016). Germ-free mice model for studying host–microbial interactions. Mouse Models for Drug Discovery: Methods and Protocols.

[108] Lundberg R, Toft MF, August B (2016). Antibiotic-treated versus germ-free rodents for microbiota transplantation studies. Gut Microbes.

[109] Farzi A, Gorkiewicz G, Holzer P (2012). Non-absorbable oral antibiotic treatment in mice affects multiple levels of the microbiota-gut-brain axis; Wiley-blackwell 111 river st, hoboken 07030-5774, NJ USA.

[110] Desbonnet L, Clarke G, Traplin A (2015). Gut microbiota depletion from early adolescence in mice: Implications for brain and behaviour. Brain Behav Immun.

[111] Ellekilde M, Selfjord E, Larsen CS (2014). Transfer of gut microbiota from lean and obese mice to antibiotic-treated mice. Sci Rep.

[112] Baldridge MT, Nice TJ, McCune BT (2015). Commensal microbes and interferon-λ determine persistence of enteric murine norovirus infection. Science.

[113] Gonzalez-Perez G, Hicks AL, Tekieli TM (2016). Maternal antibiotic treatment impacts development of the neonatal intestinal microbiome and antiviral immunity. J Immunol.

[114] Brown RL, Sequeira RP, Clarke TB (2017). The microbiota protects against respiratory infection via GM-CSF signaling. Nat Commun.

[115] Bruce-Keller AJ, Salbaum JM, Luo M (2015). Obese-type gut microbiota induce neurobehavioral changes in the absence of obesity. Biol Psychiatry.

[116] Ericsson AC, Personett AR, Turner G (2017). Variable colonization after reciprocal fecal microbiota transfer between mice with low and high richness microbiota. Front Microbiol.

[117] He B, Nohara K, Ajami NJ (2015). Transmissible microbial and metabolomic remodeling by soluble dietary fiber improves metabolic homeostasis. Sci Rep.

[118] Kelly JR, Borre Y, O'Brien C (2016). Transferring the blues: depression-associated gut microbiota induces neurobehavioural changes in the rat. J Psychiatr Res.

[119] Yano JM, Yu K, Donaldson GP (2015). Indigenous bacteria from the gut microbiota regulate host serotonin biosynthesis. Cell.

[120] Zhou D, Pan Q, Shen F (2017). Total fecal microbiota transplantation alleviates high-fat diet-induced steatohepatitis in mice via beneficial regulation of gut microbiota. Sci Rep.

[121] Ubeda C, Bucci V, Caballero S (2013). Intestinal microbiota containing Barnesiella species cures vancomycin-resistant Enterococcus faecium colonization. Infect Immun.

[122] Reikvam DH, Erofeev A, Sandvik A (2011). Depletion of murine intestinal microbiota: effects on gut mucosa and epithelial gene expression. PloS One.

[123] Grasa L, Abecia L, Forcén R (2015). Antibiotic-induced depletion of murine microbiota induces mild inflammation and changes in toll-like receptor patterns and intestinal motility. Microb Ecol.

[124] Zákostelská Z, Málková J, Klimešová K (2016). Intestinal microbiota promotes psoriasis-like skin inflammation by enhancing Th17 response. PloS One.

[125] Abt MC, Osborne LC, Monticelli LA (2012). Commensal bacteria calibrate the activation threshold of innate antiviral immunity. Immunity.

[126] Emal D, Rampanelli E, Stroo I (2017). Depletion of gut microbiota protects against renal ischemia-reperfusion injury. J Am Soc Nephrol.

[127] Kuss SK, Best GT, Etheredge CA (2011). Intestinal microbiota promote enteric virus replication and systemic pathogenesis. Science.

[128] Williams SC (2014). Gnotobiotics. Proc Natl Acad Sci USA.

[129] Fontaine CA, Skorupski AM, Vowles CJ (2015). How free of germs is germ-free? Detection of bacterial contamination in a germ free mouse unit. Gut Microbes.

[130] Hörmannsperger G, Schaubeck M, Haller D (2015). Intestinal microbiota in animal models of inflammatory diseases. ILAR J.

[131] Reyniers J, Sacksteder MR (1958). Observations on the survival of germfree C3H mice and their resistance to a contaminated environment.

[132] Gordon HA, Bruckner-kardoss E, Wostmann BS (1966). Aging in germ-free mice: life tables and lesions observed at natural death. J Gerontol.

[133] Abrams GD, Bauer H, Sprinz H (1962). Influence of the normal flora on mucosal morphology and cellular renewal in the ileum. A comparison of germ-free and conventional mice. Mount Sinai Hospial N Y.

[134] Gordon H, Wostmann B (1973). Chronic mild diarrhea in germfree rodents: a model portraying host-flora synergism. Germfree Res.

[135] Coates ME, Hewitt D, Salter D (1971). Protein metabolism in the germ-free and conventional chick. Germfree research, biological effect of gnotobiotic environments.

[136] Shimizu K, Muranaka Y, Fujimura R (1998). Normalization of reproductive function in germfree mice following bacterial contamination. Exp Anim.

[137] Wen L, Ley RE, Volchkov PY (2008). Innate immunity and intestinal microbiota in the development of Type 1 diabetes. Nature.

[138] Candon S, Perez-Arroyo A, Marquet C (2015). Antibiotics in early life alter the gut microbiome and increase disease incidence in a spontaneous mouse model of autoimmune insulin-dependent diabetes. PloS One.

[139] Hansen C, Krych L, Nielsen D (2012). Early life treatment with vancomycin propagates Akkermansia muciniphila and reduces diabetes incidence in the NOD mouse. Diabetologia.

[140] Weng M, Walker W (2013). The role of gut microbiota in programming the immune phenotype. J Dev Origins Health Dis.

[141] Jaggar M, Rea K, Spichak S (2020). You've got male: sex and the microbiota-gut-brain axis across the lifespan. Front Neuroendocrinol.

[142] Schubert AM, Sinani H, Schloss PD (2015). Antibiotic-induced alterations of the murine gut microbiota and subsequent effects on colonization resistance against *Clostridium difficile*. mBio.

[143] Zackular JP, Baxter NT, Chen GY (2016). Manipulation of the gut microbiota reveals role in colon tumorigenesis. mSphere.

[144] Rune I, Hansen C, Ellekilde M (2013). Ampicillin-improved glucose tolerance in diet-induced obese C57BL/6NTac mice is age dependent. J Diabetes Res.

[145] Atarashi K, Tanoue T, Shima T (2011). Induction of colonic regulatory T cells by indigenous Clostridium species. Science.

[146] Hansen AK (1995). Antibiotic treatment of nude rats and its impact on the aerobic bacterial flora. Lab Ani.

[147] Zhang L, Huang Y, Zhou Y (2013). Antibiotic administration routes significantly influence the levels of antibiotic resistance in gut microbiota. Antimicrob Agents Chemother.

[148] Morgun A, Dzutsev A, Dong X (2015). Uncovering effects of antibiotics on the host and microbiota using transkingdom gene networks. Gut.

[149] Ubeda C, Taur Y, Jenq RR (2010). Vancomycin-resistant Enterococcus domination of intestinal microbiota is enabled by antibiotic treatment in mice and precedes bloodstream invasion in humans. J Clin Invest.

[150] Barfod KK, Roggenbuck M, Hansen LH (2013). The murine lung microbiome in relation to the intestinal and vaginal bacterial communities. BMC Microbiol.

[151] Srinivas G, Möller S, Wang J (2013). Genome-wide mapping of gene–microbiota interactions in susceptibility to autoimmune skin blistering. Nat Commun.

[152] Fröhlich EE, Farzi A, Mayerhofer R (2016). Cognitive impairment by antibiotic-induced gut dysbiosis: analysis of gut microbiota-brain communication. Brain Behav Immun.

[153] Delungahawatta T, West C, Stanisz A (2018). A301 antibiotics increase vagal afferent firing in the mouse jejunum. J Can Assoc Gastroenterol.

[154] Delungahawatta T, Amin JY, Stanisz AM (2017). Antibiotic driven changes in gut motility suggest direct modulation of enteric nervous system. Front Neurosci.

[155] Underhill DM, Iliev ID (2014). The mycobiota: interactions between commensal fungi and the host immune system. Nat Rev Immunol.

[156] Virgin HW (2014). The virome in mammalian physiology and disease. Cell.

[157] Noverr MC, Noggle RM, Toews GB (2004). Role of antibiotics and fungal microbiota in driving pulmonary allergic responses. Infect Immun.

[158] Kim Y-G, Udayanga KGS, Totsuka N (2014). Gut dysbiosis promotes M2 macrophage polarization and allergic airway inflammation via fungi-induced PGE2. Cell Host Microbe.

[159] Hernández-Chirlaque C, Aranda CJ, Ocón B (2016). Germ-free and antibiotic-treated mice are highly susceptible to epithelial injury in DSS colitis. J Crohn's Colitis.

[160] Kuno T, Hirayama-Kurogi M, Ito S (2016). Effect of intestinal flora on protein expression of drug-metabolizing enzymes and transporters in the liver and kidney of germ-free and antibiotics-treated mice. Mol Pharm.

[161] Reinhardt C, Bergentall M, Greiner TU (2012). Tissue factor and PAR1 promote microbiota-induced intestinal vascular remodelling. Nature.

[162] Sommer F, Bäckhed F (2013). The gut microbiota—masters of host development and physiology. Nat Rev Microbiol.

[163] Josefsdottir KS, Baldridge MT, Kadmon CS (2017). Antibiotics impair murine hematopoiesis by depleting the intestinal microbiota. Blood.

[164] Iwamura C, Bouladoux N, Belkaid Y (2017). Sensing of the microbiota by NOD1 in mesenchymal stromal cells regulates murine hematopoiesis. Blood.

[165] Zhang D, Chen G, Manwani D (2015). Neutrophil ageing is regulated by the microbiome. Nature.

[166] Ichinohe T, Pang IK, Kumamoto Y (2011). Microbiota regulates immune defense against respiratory tract influenza A virus infection. Proc Natl Acad Sci.

[167] Oh JE, Kim B-C, Chang D-H (2016). Dysbiosis-induced IL-33 contributes to impaired antiviral immunity in the genital mucosa. Proc Natl Acad Sci.

[168] Naik S, Bouladoux N, Wilhelm C (2012). Compartmentalized control of skin immunity by resident commensals. Science.

[169] Luczynski P, McVey Neufeld K-A, Oriach CS (2016). Growing up in a bubble: using germ-free animals to assess the influence of the gut microbiota on brain and behavior. Interl J Neuropsychopharmacol.

[170] Uzbay T (2019). Germ-free animal experiments in the gut microbiota studies. Current Opinion in Pharmacology.

[171] Spychala MS, Venna VR, Jandzinski M (2018). Age-related changes in the gut microbiota influence systemic inflammation and stroke outcome. Ann Neurol.

[172] Lai Z-L, Tseng C-H, Ho HJ (2018). Fecal microbiota transplantation confers beneficial metabolic effects of diet and exercise on diet-induced obese mice. Sci Rep.

[173] Saiman Y, Shen TCD, Lund PJ (2021). Global microbiota-dependent histone acetylation patterns are irreversible and independent of short chain fatty acids. Hepatology.

[174] Mell B, Jala VR, Mathew AV (2015). Evidence for a link between gut microbiota and hypertension in the Dahl rat. Physiol Genomics.

[175] Sayin SI, Wahlström A, Felin J (2013). Gut microbiota regulates bile acid metabolism by reducing the levels of tauro-beta-muricholic acid, a naturally occurring FXR antagonist. Cell Metab.

[176] Wichmann A, Allahyar A, Greiner TU (2013). Microbial modulation of energy availability in the colon regulates intestinal transit. Cell Host Microbe.

[177] Fernández-Santoscoy M, Wenzel UA, Yrlid U (2015). The gut microbiota reduces colonization of the mesenteric lymph nodes and il-12-independent ifn-γ production during salmonella infection. Front Cell Infect Microbiol.

[178] Kelly CJ, Zheng L, Campbell EL (2015). Crosstalk between microbiota-derived short-chain fatty acids and intestinal epithelial hif augments tissue barrier function. Cell Host Microbe.

[179] Gopalakrishnan V, Dozier EA, Glover MS (2021). Engraftment of bacteria after fecal microbiota transplantation is dependent on both frequency of dosing and duration of preparative antibiotic regimen. Microorganisms.

[180] Barcena C, Valdés-Mas R, Mayoral P (2019). Healthspan and lifespan extension by fecal microbiota transplantation into progeroid mice. Nat Med.

[181] Cao H, Liu X, An Y (2017). Dysbiosis contributes to chronic constipation development via regulation of serotonin transporter in the intestine. Sci Rep.

[182] Routy B, Le Chatelier E, Derosa L (2018). Gut microbiome influences efficacy of PD-1–based immunotherapy against epithelial tumors. Science.

[183] Li Y, Ning L, Yin Y (2020). Age-related shifts in gut microbiota contribute to cognitive decline in aged rats. Aging.

[184] Hughes KR, Schofield Z, Dalby MJ (2020). The early life microbiota protects neonatal mice from pathological small intestinal epithelial cell shedding. FASEB J.

[185] Liu Z, Li N, Fang H (2019). Enteric dysbiosis is associated with sepsis in patients. The FASEB J.

[186] Kim SG, Becattini S, Moody TU (2019). Microbiota-derived lantibiotic restores resistance against vancomycin-resistant Enterococcus. Nature.

[187] Xu T, Ge Y, Du H (2021). Berberis kansuensis extract alleviates type 2 diabetes in rats by regulating gut microbiota composition. J Ethnopharmacol.

[188] Le Bastard Q, Ward T, Sidiropoulos D (2018). Fecal microbiota transplantation reverses antibiotic and chemotherapy-induced gut dysbiosis in mice. Sci Rep.

[189] Staley C, Kaiser T, Beura LK (2017). Stable engraftment of human microbiota into mice with a single oral gavage following antibiotic conditioning. Microbiome.

[190] Hoyles L, Fernández-Real JM, Federici M (2018). Molecular phenomics and metagenomics of hepatic steatosis in non-diabetic obese women. Nat Med.

[191] Kinnebrew MA, Ubeda C, Zenewicz LA (2010). Bacterial flagellin stimulates Toll-like receptor 5-dependent defense against vancomycin-resistant *Enterococcus* infection. J Infect Dis.

[192] Han Q, Wang J, Li W (2021). Androgen-induced gut dysbiosis disrupts glucolipid metabolism and endocrinal functions in polycystic ovary syndrome. Microbiome.

[193] Zhu Z, Kaiser T, Staley C (2021). Antibiotic conditioning and single gavage allows stable engraftment of human microbiota in mice. The Oral Microbiome.

[194] Sun X, Winglee K, Gharaibeh RZ (2018). Microbiota-derived metabolic factors reduce campylobacteriosis in mice. Gastroenterology.

[195] Guo S, Jiang D, Zhang Q (2021). Diverse role of gut microbiota on reduction of ascites and intestinal injury in malignant ascites effusion rats treated with Euphorbia kansui stir-fried with vinegar. J Ethnopharmacol.

[196] Li Q, He R, Zhang F (2020). Combination of oligofructose and metformin alters the gut microbiota and improves metabolic profiles, contributing to the potentiated therapeutic effects on diet-induced obese animals. Front Endocrinol.

[197] Ochoa-Repáraz J, Mielcarz DW, Ditrio LE (2009). Role of gut commensal microflora in the development of experimental autoimmune encephalomyelitis. J Immunol.

[198] Olson CA, Vuong HE, Yano JM (2018). The gut microbiota mediates the anti-seizure effects of the ketogenic diet. Cell.

[199] Le Roy T, Debédat J, Marquet F (2019). Comparative evaluation of microbiota engraftment following fecal microbiota transfer in mice models: age, kinetic and microbial status matter. Front Microbiol.

[200] Grant C, Loman B, Bailey M (2021). Manipulations of the gut microbiome alter chemotherapy-induced inflammation and behavioral side effects in female mice. Brain Behav Immun.

[201] Koester ST, Li N, Lachance DM (2021). Variability in digestive and respiratory tract Ace2 expression is associated with the microbiome. Plos one.

[202] Hill DA, Hoffmann C, Abt MC (2010). Metagenomic analyses reveal antibiotic-induced temporal and spatial changes in intestinal microbiota with associated alterations in immune cell homeostasis. Mucosal Immunol.

[203] Brandsma E, Kloosterhuis NJ, Koster M (2019). A proinflammatory gut microbiota increases systemic inflammation and accelerates atherosclerosis. Circ Res.

[204] Ganal SC, Sanos SL, Kallfass C (2012). Priming of natural killer cells by nonmucosal mononuclear phagocytes requires instructive signals from commensal microbiota. Immunity.

[205] Knoop KA, McDonald KG, McCrate S (2015). Microbial sensing by goblet cells controls immune surveillance of luminal antigens in the colon. Mucosal Immunol.

[206] Sougiannis A, VanderVeen B, Enos R (2019). Impact of 5 fluorouracil chemotherapy on gut inflammation, functional parameters, and gut microbiota. Brain Behav Immun.

[207] Benakis C, Brea D, Caballero S (2016). Commensal microbiota affects ischemic stroke outcome by regulating intestinal γδ T cells. Nat Med.

[208] Kim M, Galan C, Hill AA (2018). Critical role for the microbiota in CX(3)CR1(+) intestinal mononuclear phagocyte regulation of intestinal t cell responses. Immunity.

[209] Blake SJ, James J, Ryan FJ (2021). The immunotoxicity, but not anti-tumor efficacy, of anti-CD40 and anti-CD137 immunotherapies is dependent on the gut microbiota. Cell Rep Med.

[210] Brown RL, Sequeira RP, Clarke TB (2017). The microbiota protects against respiratory infection via GM-CSF signaling. Nat Commun.

[211] Zhang J, Bi JJ, Guo GJ (2019). Abnormal composition of gut microbiota contributes to delirium-like behaviors after abdominal surgery in mice. CNS Neurosci Ther.

[212] Du HX, Liu Y, Zhang LG (2020). Abnormal gut microbiota composition is associated with experimental autoimmune prostatitis-induced depressive-like behaviors in mice. Prostate.

[213] Wong SH, Zhao L, Zhang X (2017). Gavage of fecal samples from patients with colorectal cancer promotes intestinal carcinogenesis in germ-free and conventional mice. Gastroenterology.

[214] King K, Josefsdottir K, Baldridge M (2016). Antibiotics impair murine hematopoiesis by depleting intestinal microbiota.

[215] Li N, Wang Q, Wang Y (2019). Fecal microbiota transplantation from chronic unpredictable mild stress mice donors affects anxiety-like and depression-like behavior in recipient mice via the gut microbiota-inflammation-brain axis. Stress.

[216] Lendrum J, Seebach B, Klein B (2017). Sleep and the gut microbiome: antibiotic-induced depletion of the gut microbiota reduces nocturnal sleep in mice. bioRxiv.

[217] Zhang Y, Xie B, Chen X (2021). A key role of gut microbiota-vagus nerve/spleen axis in sleep deprivation-mediated aggravation of systemic inflammation after LPS administration. Life Sci.

[218] Wang M, Cao J, Gong C (2021). Exploring the microbiota-Alzheimer's disease linkage using short-term antibiotic treatment followed by fecal microbiota transplantation. Brain Behav Immun.

[219] Kim J, Kirkland R, Lee S (2020). Gut microbiota composition modulates inflammation and structure of the vagal afferent pathway. Physiol Behav.

[220] Wang Q, Wang X, Lv Y (2021). Changes in Rats' gut microbiota composition caused by induced chronic myocardial infarction lead to depression-like behavior. Front Microbiol.

[221] Shute A, Callejas BE, Li S (2021). Cooperation between host immunity and the gut bacteria is essential for helminth-evoked suppression of colitis. Microbiome.

[222] LaGamma EF, Hu F, Cruz FP (2021). Bacteria-derived short chain fatty acids restore sympathoadrenal responsiveness to hypoglycemia after antibiotic-induced gut microbiota depletion. Neurobiol Stress.

[223] Burrello C, Garavaglia F, Cribiù FM (2018). Short-term oral antibiotics treatment promotes inflammatory activation of colonic invariant natural killer t and conventional CD4(+) T cells. Front Med.

[224] Hägerbrand K, Westlund J, Yrlid U (2015). MyD88 signaling regulates steady-state migration of intestinal CD103+ dendritic cells independently of TNF-α and the gut microbiota. J Immunol.

[225] Hashiguchi M, Kashiwakura Y, Kojima H (2015). Peyer's patch innate lymphoid cells regulate commensal bacteria expansion. Immunol Lett.

[226] Thackray LB, Handley SA, Gorman MJ (2018). Oral antibiotic treatment of mice exacerbates the disease severity of multiple flavivirus infections. Cell Rep.

[227] Zhan G, Yang N, Li S (2018). Abnormal gut microbiota composition contributes to cognitive dysfunction in SAMP8 mice. Aging.

[228] Emal D, Rampanelli E, Stroo I (2017). Depletion of gut microbiota protects against renal ischemia-reperfusion injury. J Am Soc Nephrol.

[229] Guirro M, Costa A, Gual-Grau A (2019). Effects from diet-induced gut microbiota dysbiosis and obesity can be ameliorated by fecal microbiota transplantation: A multiomics approach. PLoS One.

[230] Yang C, Fang X, Zhan G (2019). Key role of gut microbiota in anhedonia-like phenotype in rodents with neuropathic pain. Transl Psychiatry.

[231] Brandl K, Plitas G, Mihu CN (2008). Vancomycin-resistant enterococci exploit antibiotic-induced innate immune deficits. Nature.

[232] Medel-Matus JS, Shin D, Dorfman E (2018). Facilitation of kindling epileptogenesis by chronic stress may be mediated by intestinal microbiome. Epilepsia Open.

[233] Steed AL, Christophi GP, Kaiko GE (2017). The microbial metabolite desaminotyrosine protects from influenza through type I interferon. Science.

[234] Yin A, Luo Y, Chen W (2019). FAM96A protects mice from dextran sulfate sodium (DSS)-induced colitis by preventing microbial dysbiosis. Front Cell Infect Microbiol.

[235] Zhao M, Xiong X, Ren K (2018). Deficiency in intestinal epithelial O-GlcNAcylation predisposes to gut inflammation. EMBO Mol Med.

[236] Ward NL, Phillips CD, Nguyen DD (2016). Antibiotic treatment induces long-lasting changes in the fecal microbiota that protect against colitis. Inflammatory Bowel Dis.

[237] Riquelme E, Zhang Y, Zhang L (2019). Tumor microbiome diversity and composition influence pancreatic cancer outcomes. Cell.

[238] Kuss SK, Best GT, Etheredge CA (2011). Intestinal microbiota promote enteric virus replication and systemic pathogenesis. Science.

[239] Hintze KJ, Cox JE, Rompato G (2014). Broad scope method for creating humanized animal models for animal health and disease research through antibiotic treatment and human fecal transfer. Gut Microbes.

[240] Goulding DR, Myers PH, Dickerson AB (2021). Comparative efficacy of two types of antibiotic mixtures in gut flora depletion in female C57BL/6 mice. Comp Med.

[241] Vétizou M, Pitt JM, Daillère R (2015). Anticancer immunotherapy by CTLA-4 blockade relies on the gut microbiota. Science.

[242] Jacobson A, Lam L, Rajendram M (2018). A Gut commensal-produced metabolite mediates colonization resistance to salmonella infection. Cell Host Microbe.

[243] Corbitt N, Kimura S, Isse K (2013). Gut bacteria drive Kupffer cell expansion via MAMP-mediated ICAM-1 induction on sinusoidal endothelium and influence preservation-reperfusion injury after orthotopic liver transplantation. Am J Pathol.

[244] Liang W, Zhao L, Zhang J (2020). Colonization potential to reconstitute a microbe community in pseudo germ-free mice after fecal microbe transplant from equol producer. Front Microbiol.

[245] Bashir ME, Louie S, Shi HN (2004). Toll-like receptor 4 signaling by intestinal microbes influences susceptibility to food allergy. J Immunol.

[246] Walsh J, Olavarria-Ramirez L, Lach G (2020). Impact of host and environmental factors on β-glucuronidase enzymatic activity: implications for gastrointestinal serotonin. Am J Physiol Gastrointest Liver Physiol.

[247] Shi Z, Zou J, Zhang Z (2019). Segmented filamentous bacteria prevent and cure rotavirus infection. Cell.

[248] Schuijt TJ, Lankelma JM, Scicluna BP (2016). The gut microbiota plays a protective role in the host defence against pneumococcal pneumonia.

[249] Gury-BenAri M, Thaiss CA, Serafini N (2016). The spectrum and regulatory landscape of intestinal innate lymphoid cells are shaped by the microbiome. Cell.

[250] Yang K, Hou Y, Zhang Y (2021). Suppression of local type I interferon by gut microbiota–derived butyrate impairs antitumor effects of ionizing radiation. J Exp Med.

[251] Johansson ME, Jakobsson HE, Holmén-Larsson J (2015). Normalization of host intestinal mucus layers requires long-term microbial colonization. Cell Host Microbe.

[252] Ye Z, Zhang N, Wu C (2018). A metagenomic study of the gut microbiome in Behcet's disease. Microbiome.

[253] Chen L, He Z, Iuga AC (2018). Diet modifies colonic microbiota and CD4+ T-cell repertoire to induce flares of colitis in mice with myeloid-cell expression of interleukin 23. Gastroenterology.

[254] Stefka AT, Feehley T, Tripathi P (2014). Commensal bacteria protect against food allergen sensitization. Proc Natl Acad Sci USA.

[255] Zhao W, Hu Y, Li C (2020). Transplantation of fecal microbiota from patients with alcoholism induces anxiety/depression behaviors and decreases brain mGluR1/PKC ε levels in mouse. Biofactors.

[256] Toubai T, Fujiwara H, Rossi C (2019). Host NLRP6 exacerbates graft-versus-host disease independent of gut microbial composition. Nat Microbiol.

[257] Mortha A, Chudnovskiy A, Hashimoto D (2014). Microbiota-dependent crosstalk between macrophages and ILC3 promotes intestinal homeostasis. Science.

[258] Oh JZ, Ravindran R, Chassaing B (2014). TLR5-mediated sensing of gut microbiota is necessary for antibody responses to seasonal influenza vaccination. Immunity.

[259] Wu X, Sun R, Chen Y (2015). Oral ampicillin inhibits liver regeneration by breaking hepatic innate immune tolerance normally maintained by gut commensal bacteria. Hepatology.

[260] Cervantes-Barragan L, Chai JN (2017). Lactobacillus reuteri induces gut intraepithelial CD4(+)CD8αα(+) T cells. Science.

[261] Ge X, Ding C, Zhao W (2017). Antibiotics-induced depletion of mice microbiota induces changes in host serotonin biosynthesis and intestinal motility. J Transl Med.

[262] Li F, Hao X, Chen Y (2017). The microbiota maintain homeostasis of liver-resident γδT-17 cells in a lipid antigen/CD1d-dependent manner. Nat Commun.

[263] Durand A, Audemard-Verger A, Guichard V (2018). Profiling the lymphoid-resident T cell pool reveals modulation by age and microbiota. Nat Commun.

[264] Adami AJ, Bracken SJ, Guernsey LA (2018). Early-life antibiotics attenuate regulatory T cell generation and increase the severity of murine house dust mite-induced asthma. Pediatr Res.

[265] Heiss CN, Mannerås-Holm L, Lee YS (2021). The gut microbiota regulates hypothalamic inflammation and leptin sensitivity in Western diet-fed mice via a GLP-1R-dependent mechanism. Cell Rep.

[266] Rakoff-Nahoum S, Paglino J, Eslami-Varzaneh F (2004). Recognition of commensal microflora by toll-like receptors is required for intestinal homeostasis. Cell.

[267] Ivanov II, Frutos Rde L, Manel N (2008). Specific microbiota direct the differentiation of IL-17-producing T-helper cells in the mucosa of the small intestine. Cell Host Microbe.

[268] Kim SH, Cho BH, Kiyono H (2017). Microbiota-derived butyrate suppresses group 3 innate lymphoid cells in terminal ileal Peyer's patches. Sci Rep.

[269] Diehl GE, Longman RS, Zhang JX (2013). Microbiota restricts trafficking of bacteria to mesenteric lymph nodes by CX(3)CR1(hi) cells. Nature.

[270] Balmer ML, Schürch CM, Saito Y (2014). Microbiota-derived compounds drive steady-state granulopoiesis via MyD88/TICAM signaling. J Immunol.

[271] Park JH, Kotani T, Konno T (2016). Promotion of Intestinal Epithelial Cell Turnover by Commensal Bacteria: Role of Short-Chain Fatty Acids. PLoS One.

[272] Vaishnava S, Behrendt CL, Ismail AS (2008). Paneth cells directly sense gut commensals and maintain homeostasis at the intestinal host-microbial interface. Proc Natl Acad Sci USA.

[273] Ichinohe T, Pang IK, Kumamoto Y (2011). Microbiota regulates immune defense against respiratory tract influenza A virus infection. Proc Natl Acad Sci USA.

[274] Ismail AS, Severson KM, Vaishnava S (2011). Gammadelta intraepithelial lymphocytes are essential mediators of host-microbial homeostasis at the intestinal mucosal surface. Proc Natl Acad Sci USA.

[275] Schnepf D, Hernandez P, Mahlakõiv T (2021). Rotavirus susceptibility of antibiotic-treated mice ascribed to diminished expression of interleukin-22. PloS One.

[276] Yoshiya K, Lapchak PH, Thai T-H (2011). Depletion of gut commensal bacteria attenuates intestinal ischemia/reperfusion injury. Am J Physiol Gastrointest Liver Physiol.

[277] Yan J, Herzog JW, Tsang K (2016). Gut microbiota induce IGF-1 and promote bone formation and growth. Proc Natl Acad Sci USA.

[278] Sui H, Zhang L, Gu K (2020). YYFZBJS ameliorates colorectal cancer progression in Apc Min/+ mice by remodeling gut microbiota and inhibiting regulatory T-cell generation. Cell Commun Signaling.

[279] Platt DJ, Lawrence D, Rodgers R (2021). Transferrable protection by gut microbes against STING-associated lung disease. Cell Rep.

[280] Khosravi A, Yáñez A, Price JG (2014). Gut microbiota promote hematopoiesis to control bacterial infection. Cell Host Microbe.

[281] Zhang D, Chen G, Manwani D (2015). Neutrophil ageing is regulated by the microbiome. Nature.

[282] Naik S, Bouladoux N, Wilhelm C (2012). Compartmentalized control of skin immunity by resident commensals. Science.

[283] Sun Z, Li J, Dai Y (2020). Indigo naturalis alleviates dextran sulfate sodium-induced colitis in rats via altering gut microbiota. Front Microbiol.

[284] Zákostelská Z, Málková J, Klimešová K (2016). Intestinal microbiota promotes psoriasis-like skin inflammation by enhancing Th17 response. PLoS One.

[285] Wang S, Huang M, You X (2018). Gut microbiota mediates the anti-obesity effect of calorie restriction in mice. Sci Rep.

[286] de la Visitacion N, Robles-Vera I, Toral M (2021). Gut microbiota contributes to the development of hypertension in a genetic mouse model of systemic lupus erythematosus. Bri J Pharmacol.

[287] Sawa S, Lochner M, Satoh-Takayama N (2011). RORγt+ innate lymphoid cells regulate intestinal homeostasis by integrating negative signals from the symbiotic microbiota. Nat Immunol.

[288] Zhang Y, Huang R, Cheng M (2019). Gut microbiota from NLRP3-deficient mice ameliorates depressive-like behaviors by regulating astrocyte dysfunction via circHIPK2. Microbiome.

[289] Atarashi K, Nishimura J, Shima T (2008). ATP drives lamina propria T(H)17 cell differentiation. Nature.

[290] Levy M, Thaiss CA, Zeevi D (2015). Microbiota-modulated metabolites shape the intestinal microenvironment by regulating NLRP6 inflammasome signaling. Cell.

[291] Kernbauer E, Ding Y, Cadwell K (2014). An enteric virus can replace the beneficial function of commensal bacteria. Nature.

